# Pemphigus Vulgaris: Short Time to Relapse in Patients Treated in a Danish Tertiary Referral Center

**DOI:** 10.3389/fmed.2019.00259

**Published:** 2019-11-29

**Authors:** Aheen Faisal Mohamad, Lars Iversen, Rikke Bech

**Affiliations:** ^1^Aalborg University Hospital, Aalborg, Denmark; ^2^Department of Dermatology, Aarhus University Hospital, Aarhus, Denmark

**Keywords:** prednisolone, mortality, comorbidity, treatment, pemphigus vulgaris

## Abstract

Pemphigus vulgaris is an autoimmune skin disorder with development of blisters in the skin and mucosa, and it can be a life-threatening disease if not treated. Corticosteroids have been a cornerstone for treating PV, but because of side effects the treatment is combined with other conventional immune modulating drugs and rituximab. The Danish treatment protocol for pemphigus vulgaris is similar to the other Scandinavian countries, and therefore this study is of importance for clinicians in the Scandinavian countries as well as other European countries. We retrospectively identified all patients with Pemphigus vulgaris in our tertiary center over a 7-year period in order to register patient characteristics, treatment, adverse events, comorbidities and the effect of prednisolone dose on remission. In this study 19 patients met the inclusion criteria and remission was seen after a mean of 19.9 weeks, and relapse was seen in 50% after the mean time of 15 weeks. Time to relapse in our study is relatively short compared to studies in which rituximab is used as a first-line drug in treating pemphigus vulgaris.

## Introduction

Pemphigus vulgaris (PV) is an autoimmune skin disorder with development of blisters in the skin and in mucosa. PV is caused by autoantibodies attacking desmoglein 1 (Dsg1) and 3 (Dsg3), which are responsible for cell-cell adhesion. Binding of autoantigens to the intercellular connections leads to disruption of cell-cell adhesion, called acantholysis (1, 2).

Clinical features of PV consist of blisters in the oral cavity and cutaneous flaccid blisters that easily rupture, leaving the skin with painful erosions that easily become infected (3). Immunofluorescence is used to detect immunoglobulin (IgG) or C3 deposition on the basement membrane.

PV is a chronic disorder and can be fatal if not treated (3). Systemic treatment with high doses of corticosteroids is regularly given. Because of the severe side effects of corticosteroids, treatment is combined with other immune modulating drugs and/or rituximab to keep the steroid dose as low as possible (1, 4–6).

## Background

From former studies of bullous pemphigoid (7), we know that high dose prednisolone (>45 mg/day) treatment is associated to longer admission time. However, no association between high/low corticosteroid dose and remission rate, relapse rate and treatment length was found. We considered whether corticosteroid treatment regimen was associated to remission, relapse and/or comorbidities in patients with pemphigus vulgaris? Therefore, the aim of the present study was to record total doses of prednisolone given to patients with PV either solely or in combination with other immunosuppressants. Additionally, comorbidities acquired before, during, or after diagnosis of PV were listed and a mortality rate was calculated (8–10).

## Methods

The medical records were retrieved for all patients admitted with a diagnosis of PV at the Department of Dermatology and Venereology at Aarhus University Hospital, Aarhus, Denmark, in the timespan 2011–2018. The project was approved by the Danish Data Protection Agency. Patients were included if they had had a biopsy that verified a diagnosis of PV according to the Danish pathology database, Patoweb. Patients younger than 18 years were excluded. A biopsy was considered diagnostic for PV if direct immunofluorescence showed deposits of IgG or C3 on the basement membrane.

Treatment was based on the department of dermatology's guidelines for patients with PV.

SPSS package software was used for statistical analysis. Graphpad Prism and Excel were used for figures. The descriptive analysis is made on the basis of continuous variables given as means with 95% confidence intervals (CI) and standard deviations (SDs) and categorical variables given in percentages. Treatment outcomes were categorical and compared using *p*-values computed from Fisher's exact test. *p*-values < 0.05 were considered statistically significant.

## Results

In the period 2011–2018, 64 patients were identified, and 19 of these fulfilled the inclusion criteria. Ten patients were excluded because they appeared twice. A total of 15 patients did not fulfill the diagnostic criteria for PV and were all excluded. Due to limited access to patients' medical records before 2011, this research was limited to the time period 2011–2018. Nineteen patients were excluded because they were diagnosed with PV before 2011. Furthermore, one patient diagnosed with PV was below 18 years of age and was therefore excluded.

The age of PV onset ranged from 21 to 101 years, with a mean of 57.8, 95% CI (48.4–67.3), SD 21.06. Female: male ratio was 0.82. Five patients were of other ethnic origin than Danish, 14 patients were ethnically Danish. There was no significant difference in age at PV onset between male and female patients (56.3 ± 20.7 vs. 60.0 ± 22.8 years, respectively; *p* = 0.7).

A DXA (dual-energy X-ray absorptiometry) was performed in 9/19 patients. Four patients developed osteopenia and one patient was diagnosed with osteoporosis during the first 12-month period (Figure 2). Seven patients never had a DXA scan even though they met the national guideline criteria for being in a special risk category for developing osteoporosis. At the time of diagnosis of PV nine patients had no comorbidities but two of them developed osteopenia. Two patients did not receive any prednisolone and one patient died within 2 months of diagnosis (Supplementary Figure 1).

One patient became diabetic and had many prednisolone side effects, including moon face, buffalo hump, and myopathy. The same side effects appeared in another patient, presumably caused by very high doses of prednisolone.

Some of the patients had comorbidities before being diagnosed with PV, see Table 1. Three patients had hypertension, and two of these also had hypercholesterolemia. One patient had chronic heart failure, one had aorta insufficiency, one had migraine, and one patient had epilepsy. Two patients had previously been treated for cancer: one for breast cancer and the other for colorectal cancer. Two of the nineteen (2/19) PV patients were treated with ACE inhibitors (Enalapril) at PV diagnosis. ACE inhibitors are known to be able to elicit or maintain PV. However, one of the two patients discontinued Enalapril when PV had been diagnosed. Yet, the PV disease was unaffected by the discontinuation of ACE inhibitor in this patient.

**Table 1:** Treatment specifications and comorbidities.

**Treatment**	**Patients**
Oral prednisolone	17
Oral prednisolone and rituximab	13
Oral prednisolone and azathioprine	7
Oral prednisolone and MTX	3
Oral prednisolone and dapsone	2
Oral prednisolone and plasmapheresis	2
Comorbidities	
Hypertension	3
Hypercholesterolemia	2
Chronic heart failure	1
Aorta insufficiency	1
Migraine	1
Epilepsy	1
Dermatitis herpitiformis	1
Celiac disease	1
Breast cancer	1
Colorectal cancer	1

Prednisolone was the most widely used treatment. Seventeen of the 19 patients received prednisolone. One patient refused to receive prednisolone because of obesity and was treated with cyclosporine, and the other patient was well treated on azathioprine and topical corticosteroids. The remaining 17 patients received prednisolone along with other adjuvants.

**Figure 1:**
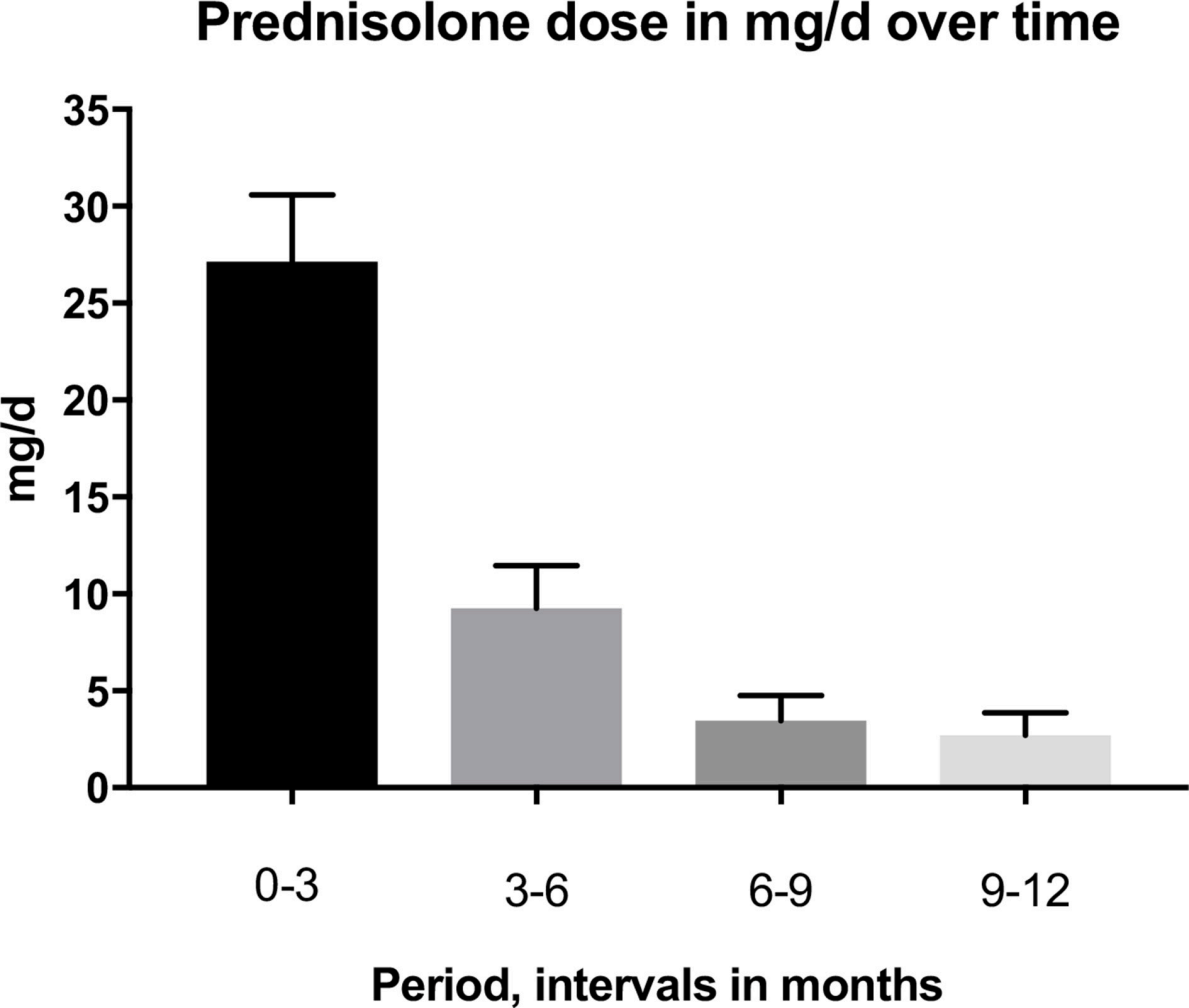
Mean dose prednisolone per interval with the standard error of mean SEM.

The primary treatment regimen was prednisolone and azathioprine, some were also given rituximab later in their course of treatment. Rituximab was used only for recalcitrant patients, for patients with very severe PV disease and for patients with contraindications to other immunosuppresants, e.g., patients with a history of malignant disease. The mean prednisolone doses (mg/d) were at baseline 0–3 months 27.15 mg/d SD 14.9 (19 patients), 3–6 months 9.26 mg/d SD 9.3 (18 patients), 6–9 months 3.46 mg/d SD 5.4 (18 patients), and 9–12 months 2.71 mg/d SD 49 (18 patients). A comparison of baseline prednisolone doses to the other time periods revealed that the dose of prednisolone decreased significantly over time. The changes in prednisolone dose are shown in Figure 1.

**Figure 2:**
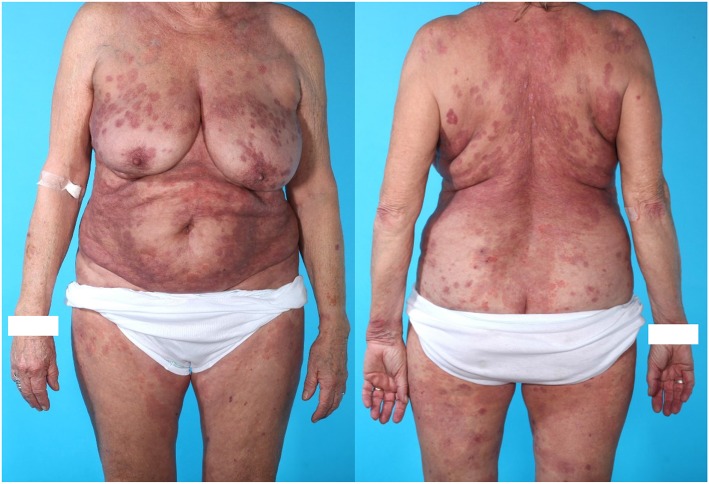
Seventy six years old (at diagnosis of PV) ethnically danish woman with mucocutaneous PV, celiac disease, and former dermatitis herpetiformis as well as essential hypertension. The patient did not receive treatment with ACE inhibitor. Skin biopsy showed acantholysis. DIF on the skin biopsy showed intercellular deposition of IgG. The patient was treated with oral prednisolon, Methotrexate and two times Rituximab. Time to remission was 20.7 weeks which is close to mean time to remission (19.9 weeks) in the 19 included patients. The patient received a total dose of 2,495 mg prednisolone, which placed her in the “low dose prednisolone” group. This patient was later diagnosed with osteoporosis on DXA scan.

In the 19 included PV patients, none were exclusively mucosal dominant. Four patients had cutaneous PV, and 14 patients had mucocutaneous PV. Most of the patients (10/19) with mucocutaneous PV had affection of skin and oral mucosa. Three patients with mucocutaneous PV had affection of skin, oral mucosa, and genital (vagina or penis, respectively) mucosa.

No patients had deposition of C3 only in DIF. Eight patients had IgG only in DIF, seven of these DIFs were done on a skin biopsy and two were done on a mucosal biopsy from gingiva and vagina, respectively. Five patients had C3 as well as IgG deposition in DIF on a skin biopsy, and one patient had C3 and IgG deposition in DIF on a mucosal biopsy from gingiva. In only one patient of the nineteen included patients DIF was done on both a skin and a mucosal biopsy. In this patient DIF showed IgG deposition on the skin biopsy but no specific reactions in DIF on mucosal (gingiva) biopsy. For additional information of DIF data, please see Supplementary Table 1.

Almost every patient started treatment with an adjuvant within the 1st week after onset of prednisolone treatment or 1 week later. Rituximab (1,000 mg) was given as an I:V: infusion two times at an interval of 2 weeks. This treatment was initiated after approximately 2 months of treatment with prednisolone and other adjuvants.

One patient had recalcitrant PV and was also treated with plasmapheresis once a month. Despite this, the patient never achieved remission.

By dividing patients into two groups, treatment outcomes could be compared. The groups were classified as receiving more than or <3,782 mg prednisone (mean total dose) during the first 12 months of treatment. Treatment outcome was measured as remission or no remission during the 12 months of follow-up time. Remission was defined as healing of blisters and no appearance of new blisters. The *p*-value was >0.05 and therefore prednisolone dose did not have any statistically significant effect on remission.

Remission was achieved in 16 patients at a mean of 19.9 weeks. Relapse was defined as appearance of new bullae. Remission followed by relapse was seen in nine patients (50%). We calculated mean time to relapse for the whole studied group (19 patients). The mean time from remission to the appearance of new bullae (relapse) was 15 weeks.

Minor adverse events like scalp infection, candida albicans in the oral cavity, diarrhea, urinary tract infection, and skin infection with *Staphylococci aureus* were seen in 9/19 (47%) patients during the first 12-month period.

Three patients (16%) had major adverse events. One patient had a single incidence of pneumonia. Another had pneumonia followed by septicemia, and a third patient had a reactivation of herpes zoster followed by pneumonia and septicemia and died. Thus, the mortality rate among our patients with PV was 5.3% (1/19) during the first 12 months of follow-up. The mortality rate was calculated to be 37 patients per 1,000 person years.

A PDAI score was found for 18 of the 19 patients. For one patient, it was not possible because of the poor quality of the description in the medical record. The majority of patients had a moderate PV according to PDAI score. Four patients had a significant PV, and only one had an extensive disease.

Results did not show a significant correlation between PDAI status and prednisolone dose.

## Discussion

We found no association between prednisolone dose (mean total dose more than or <3,782 mg) and time to remission. The patients in this study were down-regulated in prednisolone dose if they experienced clinical improvement. This makes remission and prednisolone dose dependent variables, and can explain why no association between the two variables was found. A prospective study in which patients are randomized to either low- or a high-dose prednisolone treatment could contribute with valuable information on prednisolone's role in inducing remission.

The mean time to relapse was relatively short in this study compared to other studies of patients with PV. This might be due to the fact that Prednisolone treatment was tapered too fast and before the patient achieved maximum effect of the Rituximab treatment. It is our clinical experience that most patients do not achieve maximum effect of the Rituximab infusion until 8–12 weeks after Rituximab infusion.

We attempted to determine the severity of PV disease by calculating PDAI based on information from the patient record (11). The ability to perform precise PDAI ranging was difficult. In a few cases, the clinician had thoroughly described size, number, and placement of the blisters. But in the vast majority, the information lacked precise details on location and total number of the blisters. In particular, blisters in the oral cavity were not described as located to a particular region of the oral cavity. This complicated the ranging of patients according to the PDAI and may possibly have underestimated the real PDAI score. In a prospective study, clinicians can be educated to register objective findings and comorbidities precisely so that the clinical data can be used in research.

The strengths of this study are the long study period and the validation of all PV diagnoses with direct immunofluorescence. The Danish civil registration number register in Denmark ensures that all procedures, administered medicine, and consultations are systematically documented. Furthermore, all patients' medical records were reviewed individually.

Selection bias may have had an influence on outcomes because the most severe cases in the region are referred to the Department of Dermatology and Venereology at Aarhus University Hospital. Also, the lack of patients with exclusive mucosal PV in this study group might reflect that these patients may primarily be referred to other departments in the hospital such as the oral surgeon unit.

The limitation of this study was the small sample size, hence the limited use of statistics. It should be taken into consideration that this is a retrospective study; the associations are therefore not conclusive.

Tavakolpour et al. report that relapse of disease is expected 6–10 months after infusion of rituximab (12). In this study, we found that 50% of the patients had a relapse after a mean of 15 weeks. This is a markedly shorter time period than expected. Relapse was assessed on clinical features alone and by different clinicians every time. In the future, it could be intriguing to also observe the serologic response in the same way as Horváth et al. did. In Horváth et al.'s study CD20+ B cells and specific antibodies against Dsg1 and Dsg3 were measured at the beginning of therapy and at every scheduled visit. Horváth et al. showed that a single course of two infusions of rituximab (500 mg each) at an interval of 2 weeks had a great effect on B-cell number and less on Dsg3 levels (13).

Knowledge of the serologic aspects of PV could clarify the disease process and elucidate how remission is accomplished and maintained.

In a multiprotocol for treatment of patients with pemphigus vulgaris or pemphigus foliaceus, Grando showed that repeated cycles with immunoglobulin (IVIg) infusion and Rituximab treatment combined with mitochondrion protecting drugs resulted in rapid disease control (0.2 months), short time to remission (1.7 months), and long time to relapse (mean time to relapse off drugs, 15.8 months). This seems to be a very efficient and safe treatment regimen for pemphigus vulgaris. However, the treatment is very expensive and hence will not be applicable to all patients or health care systems. In comparison the mean time to relapse in our study group was 15 weeks (14).

A study by Kanwar et al. investigated the clinical and serological difference between patients receiving two doses of 1,000 mg rituximab or 500 mg rituximab 14 days apart. Their results showed a statistically significant decline in Dsg1 and Dsg3 antibodies and a greater fall in clinical scoring in the group receiving 1,000 mg rituximab (15).

The above-mentioned studies enhance the serologic effect of rituximab on PV. Joly et al. showed a significant reduction in prednisolone dose in patients treated with rituximab (4). The patients in the study by Joly et al. had recalcitrant PV. Results showed that a single cycle of rituximab decreased the prednisolone dose required from 94 to 12 mg per day. Patients in this study were down-regulated in prednisolone dose to approximately 3.46 mg/d after only 6 months. This dose is considered a low dose with limited side effects.

In this study, one patient had recalcitrant PV and was treated with plasmapheresis once a month along with other adjuvants. Despite this, the patient never achieved remission. Kridin et al. states that immunoadsorption is superior to plasmapheresis. Plasmapheresis unselectively and inadvertently removes all plasma proteins in contrast to immunoadsorption, which removes circulating IgG autoantibodies (7).

In the study by Pfütze et al. patients treated with immunoadsorption had lower Dsg1 and Dsg3 antibody titres and a decrease in both skin and mucosal ABSIS (Autoimmune Bullous Skin Disorder Intensity Score). ABSIS and PDAI are the most often used scoring system to determine PV severity (11). In Pfütze's study, prednisolone was also reduced within 12 months (16). This points at immunoadsorption as a relevant treatment option for patients with recalcitrant PV. There are, however, some disadvantages associated with immunoadsorption because it is expensive and not generally available.

## Conclusion

In conclusion, patients in this study showed clinical improvement and remission after a mean period of 19.9 weeks. Remission did not seem to correlate with prednisolone dose. The time to relapse (15 weeks) was relatively short in the studied PV group compared to other studies. This calls for a revision of our treatment guidelines in favor of more liberal use of Rituximab and IVIg treatment. The PV treatment in this study was primarily a combination of prednisolone and azathioprine or rituximab. Prednisolone was reduced considerably after 12 months, and consequently the risk of side effects was lowered over time. DXA scans must be used systematically in the clinical setting to reduce the risk of prednisolone-induced osteoporosis in patients with PV.

## Data Availability Statement

All datasets generated for this study are included in the article/Supplementary Material.

## Ethics Statement

This study was conducted in accordance with the World Medical Association's Declaration of Helsinki. We consulted the national (Datatilsynet, Danmark) and local ethics committee (Styrelsen for Patientsikkerhed, Region Midt) and learned that the study was exempt from ethics approval and written consent from the included patients. This exemption from ethics approval was motivated by the fact that the study was retrospective, based on journal information and without identifiable-to-a-person data, and thus the study was considered a quality assurance study.

## Author Contributions

AM did the initial data collection, statistical analysis, and wrote the first manuscript draft. LI contributed to design of the study and all revisions of the manuscript. RB assisted AM in all phases of data collection, statistical analysis, the writing process, and revisions of the manuscript.

### Conflict of Interest

The authors declare that the research was conducted in the absence of any commercial or financial relationships that could be construed as a potential conflict of interest.

## Abbreviations

PV: Pemphigus vulgaris
IF: Direct immunofluorescence
Dsg1: Desmoglein 1
Dsg3: Desmoglein 3
PDAI: Pemphigus Disease Area Index
SD: Standard deviation
CI: Confidence interval
Pt: Patient
DXA: Dual-energy X-ray absorptiometry
SEM: Standard error of mean
mg/d: milligrams per day
ABSIS: Autoimmune Bullous Skin Disorder Intensity Score.
