# Supplementary material for: Pemphigus Vulgaris: Short Time to Relapse in Patients Treated in a Danish Tertiary Referral Center
Source: Front Med (Lausanne). 2019 Nov 29;6:259. doi: 10.3389/fmed.2019.00259 (PMC6896223; doi:10.3389/fmed.2019.00259)
Supplement: Supplementary file 1 [file Table_1.pdf]

Supplementary Table 1

Patient no	Date of birth	Deposition in DIF	Affection skin/mucosa	ACE inhibitor?
2	010846	IgG (skin)	Skin	No
3	020376	IgM (skin)	Skin	No
4	050478	IgG (skin)	Skin and larynx	No
5	050776	IgA, IgG (skin)<br>IgG (oral mucosa)	Skin and oral cavity	No
6	090576	IgA, IgG (oral mucosa)	Skin and oral cavity	No
7	100933	IgG, IgM, C3 (skin)	Skin and oral cavity	Yes (Enalapril)
8	101061	IgG, C3 (oral cavity)	Skin and oral cavity	No
9	110355	IgA, IgG (skin)	Skin and oral cavity	No
10	170225	IgA, C3	Skin	No
11	190992 (excluded because of age < 18 years)	IgG	Skin	No
12	191136	IgG (skin)<br>IgA, IgG, C3 (oral cavity)	Skin and oral cavity	No
13	250915	IgG	Skin	No
14	200841	IgG (skin)	Skin and oral cavity	No
15	220541	IgG	Skin	Yes (Enalapril)
16	240862	IgG, C3 (skin)	Skin and oral cavity	No
17	241199	IgG (vulva)	Skin, oral cavity and vulva	No
18	260648	IgG, C3	Skin	No
19	271174	IgG (oral mucosa)	Skin, oral cavity and penis	No
20	310167	IgG, C3 (skin)	Skin and oral cavity	No
21	310172	IgG, C3 (skin)	Skin and oral cavity	No
