# Supplementary material for: Pemphigus Vulgaris: Short Time to Relapse in Patients Treated in a Danish Tertiary Referral Center
Source: Front Med (Lausanne). 2019 Nov 29;6:259. doi: 10.3389/fmed.2019.00259 (PMC6896223; doi:10.3389/fmed.2019.00259)
Supplement: Supplementary file 2 [file Data_Sheet_1.pdf]

Supplementary

Figure I: Quantitative measurement of patients having a DXA scan or not. Outcome of DXA scan in different colors.

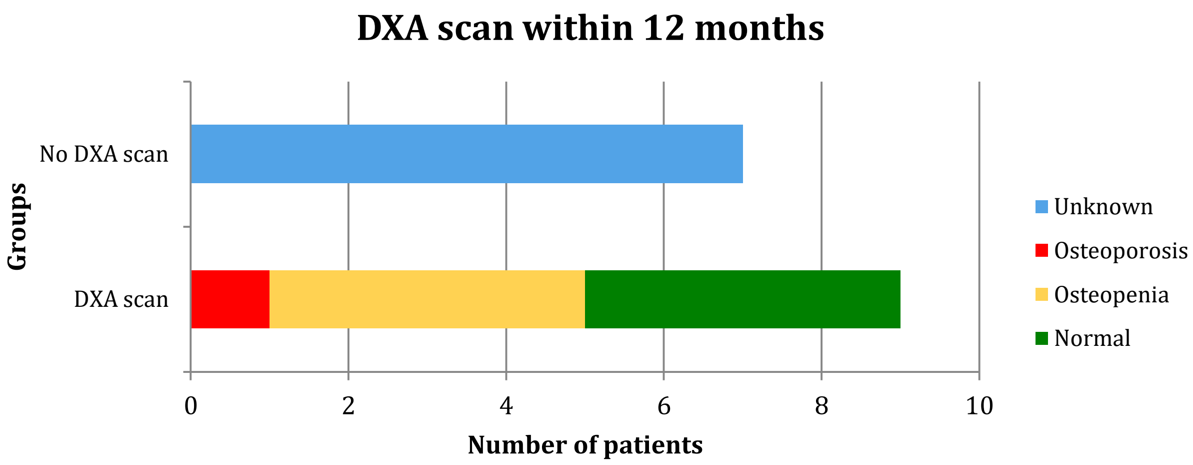

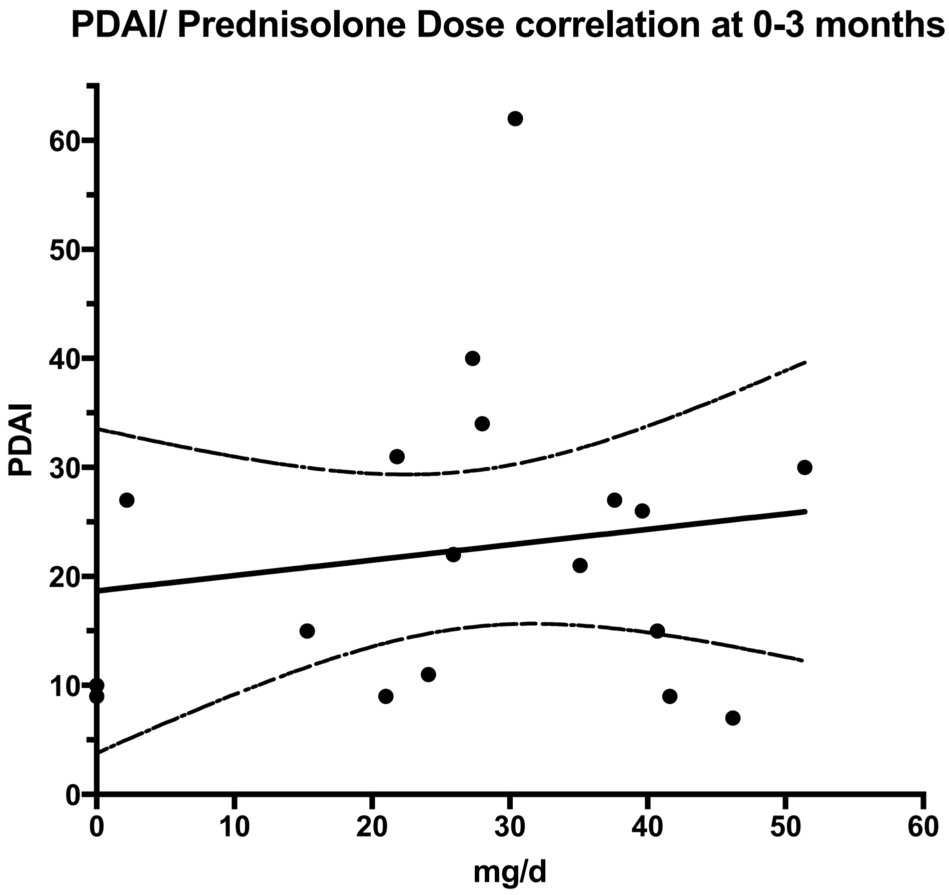

Figure II: PDAI score and mean prednisolone dose at 0–3 months for each patient. Dotted line represents 95% CI.
